# Novel late-stage radiosynthesis of 5-[18F]-trifluoromethyl-1,2,4-oxadiazole (TFMO) containing molecules for PET imaging

**DOI:** 10.1038/s41598-021-90069-x

**Published:** 2021-05-21

**Authors:** Nashaat Turkman, Daxing Liu, Isabella Pirola

**Affiliations:** 1 Department of Radiology, School of Medicine, State University of New York at Stony Brook, Long Island, Stony Brook, NY 11974 USA; 2 Stony Brook Cancer Center, Long Island, Stony Brook, NY USA

**Keywords:** Synthetic chemistry methodology, Diagnostic markers

## Abstract

Small molecules that contain the (TFMO) moiety were reported to specifically inhibit the class-IIa histone deacetylases (HDACs), an important target in cancer and the disorders of the central nervous system (CNS). However, radiolabeling methods to incorporate the [18F]fluoride into the TFMO moiety are lacking. Herein, we report a novel late-stage incorporation of [18F]fluoride into the TFMO moiety in a single radiochemical step. In this approach the bromodifluoromethyl-1,2,4-oxadiazole was converted into [18F]TFMO via no-carrier-added bromine-[18F]fluoride exchange in a single step, thus producing the PET tracers with acceptable radiochemical yield (3–5%), high radiochemical purity (> 98%) and moderate molar activity of 0.33–0.49 GBq/umol (8.9–13.4 mCi/umol). We validated the utility of the novel radiochemical design by the radiosynthesis of [18F]TMP195, which is a known TFMO containing potent inhibitor of class-IIa HDACs.

## Introduction

The human histone deacetylases (HDACs) form a large family of 18 members and are catogerized into four classes (I–IV)^1,2^. The class-IIa HDACs is a sub family comprised of four enzymes: HDAC-4, HDAC-5, HDAC-7 and HDAC-9^3–5^. There is mounting evidence on a key role for the class-IIa HDACs in various cancers^6–11^ and in the disorders of the central nervous system (CNS)^12–16^ including memory and cognitive impairment, dementia and behavioral changes among others^17–25^.

[^11^C]martinostat was the first successful class-I HDAC (HDAC1, 2 and 3) inhibitor-based tracer^26–28^. The same group also reported PET imaging in rodent, nonhuman primate and clinical validation in human brain with a brain-penetrant and semi-selective HDAC6 inhibitor^29,30^. However, reliable class-IIa HDAC PET tracers for imaging of cancer and the disorders of the CNS are lacking. Therefore, we targeted class-IIa HDACs for PET tracer development.

**Figure 1:**
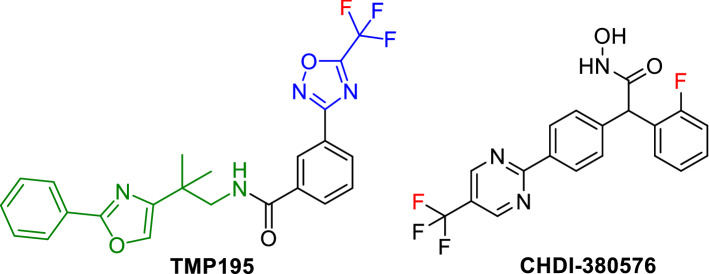
Reported highly potent inhibitors of class-IIa HDACs^31,34^.

TMP195 and CHDI-390576 (Fig. 1) are among the very few potent and highly specific class-IIa HDAC inhibitors that were recently reported in the literature^31,32^ and followed by a plethora of class-IIa HDAC inhibitors that were disclosed in patent applications. CHDI-390576 is a second generation benzhydryl hydroxamic acid with improved CNS properties and selectivity to class-IIa HDACs over the previously reported cyclopropane hydroxamic acids by the same group^33^.

We selected the 5-trifluoromethyl-1,2,4-oxadiazole (TFMO) moiety of TMP195 for radiotracer development. Considering that both CHDI-390576 and TMP195 contain the trifluoromethyl moiety, we theorize that the TFMO moiety is more accessible for late-stage radiolabelling with [18F]fluoride. We surmised that the bromodifluoromethyl-1,2,4-oxadiazole analogue (labelling bromo-precursor, i.e. compound **23**) can be easily generated by swapping the trifluoroacetic anhydride (**18**) with bromodifluoroacetic anhydride (**9**) in the final synthetic step preceding the radiofluorination reaction. Another major advantage of this novel radiolabeling strategy is that the bromodifluoromethyl-1,2,4-oxadiazole can facilitate a single late-stage radiolabelling step to incorporate the [18F]fluoride into the [^18^F]TFMO via a simple bromine to [^18^F]fluoride exchange. Moreover, this practical synthesis allows for facile generation of library of radiolabled TFMO containing molecules using a single synthetic approach as demenostrated by several examples in this work including the radiofluorination of [^18^F]TMP195 in a single step.

Notably, various strategies for incorporating the [18F]fluoride into the trifluoromethyl moiety has been described ^35^. However, producing [18F]trifluoromethyl-containing PET tracers in one pot and in high specific activity remains a fundamental challenge^36^. Early reports on the synthesis of [18F]trifluoromethyl-containing PET tracers were performed via carrier added [^18^F]-^19^F isotopic exchange at high temperature^37,38^. However, the carrier added radiolabeling methods were hampered by isotopic dilution with 19F-fluoride. This is detrimental to utility of such tracers for PET imaging since the nonradioactive fraction is predominant in the final product leading to excessive self-blocking. To overcome this limitation, classical direct nucleophilic radiofluorination via [^18^F]-for-Br nucleophilic substitution was utilized to obtain [^18^F]trifluoromethyl arenes under no-carrier-added conditions^39^. However, the multistep radiosynthesis and the presence of inseparable labelling precursor confounded the specific activity. An efficient, Cu-mediated coupling of difluoroiodomethane with aryl iodides for the radiosynthesis of [^18^F]trifluoromethyl arenes was recently reported^40^. However, this radiofluorination is limited to aryl iodides precursors and the use of low boiling difluoroiodomethane starting material likely will further hamper the wide utility of this method. An efficient no-carrier-added, however, multicomponent protocol for facile [^18^F]trifluoromethylation of aromatic and heteroaromatic systems using (hetero)aryl iodide, and [^18^F]CF_3_Cu generated in situ from methyl chlorodifluoroacetate, CuI and TMEDA was recently reported to generate high radiochemical yield and moderate molar activity of 0.1 GBq/μmol (2.7 mCi/uM)^36^.

In contrast to the above reports, the radiofluorination reported here is a straightforward late stage, performed in a single radiochemical step via [^18^F]-for-Br exchange under no-carrier-added conditions which led to tracers with high radiochemical purity and relatively higher molar activity of 0.33–0.49 GBq/umol (8.9–13.4 mCi/umol) when compared to the previously reported molar activity values for the [18F]trifluoromethyl-containing PET tracers. Moreover, the unreacted labelling bromo-precursor (starting material in large excess) is separable and did not confound the specific activity (Fig. 1, Sl). Also, we experimentally demonstrated that the [^18^F]-^19^F isotopic exchange does not occur and as such does not contribute to the radioactive product, and consequently does not influence the molar activity. Moreover, we expect the molar activity to improve significantly once starring with higher radioactivity using an onsite cyclotron for [18F]fluoride production. Currently, we are purchasing the [18F]fluoride from an outside source and it undergoes significant decay (> 2 half-lives) prior to the start of our experiments. Importantly, our radiolabelling approach can be widely applicable to develop new generation of TFMO-containing molecules without changing method of preparation for bromodifluoromethyl-1,2,4-oxadiazole, radiolabelling procedure or reaction conditions.

## Results and discussion

**Figure 2:**
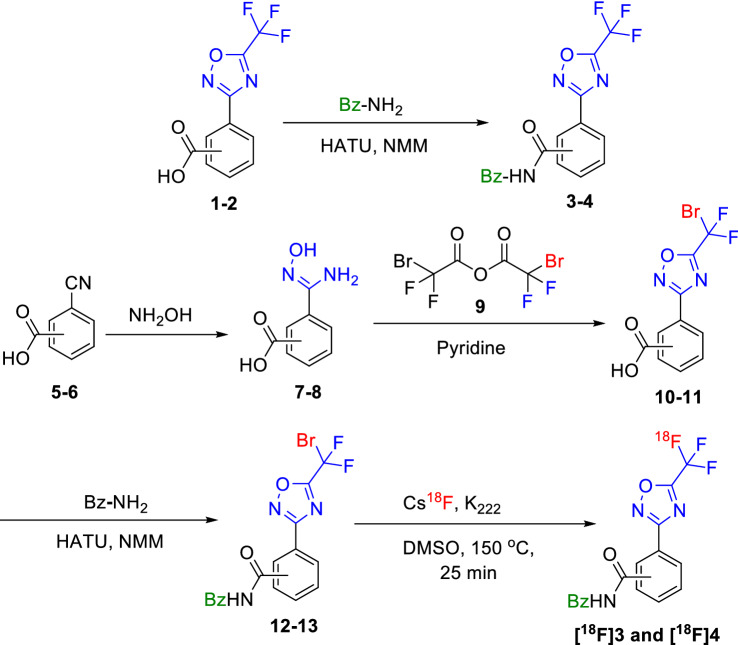
Radiosynthesis of ^18^F-TFMO containing amides.

**Figure 3:**
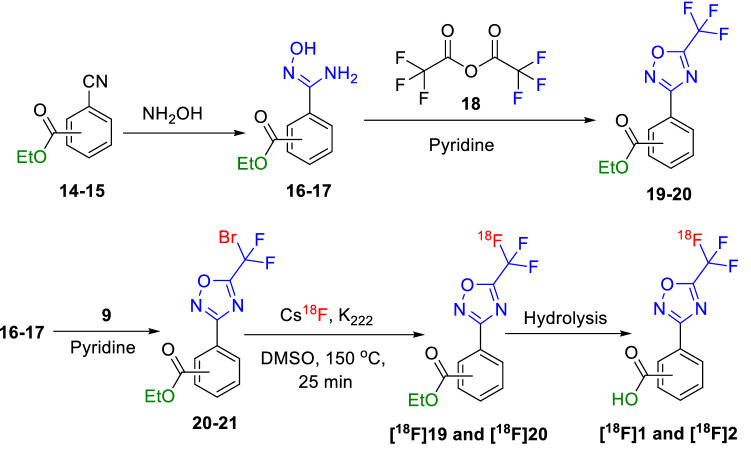
Radiosynthesis of ^18^F-TFMO containing esters and benzoic acids.

**Figure 4:**
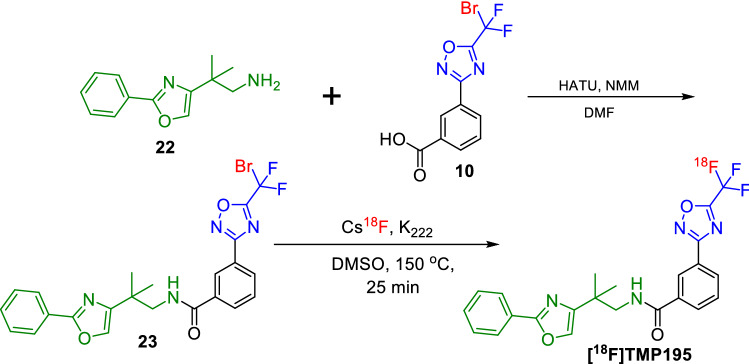
Radiosynthesis of [^18^F]TMP195.

Given our specific interest in targeting the class-IIa HDACs for PET tracer development, the TFMO bearing molecules presented an attractive target for PET tracer development. The TFMO is a distinctive class-IIa HDAC pharmacophore motif that interacts with the zinc ion at the bottom of the class-IIa HDAC catalytic pocket rendering high specificity and selectivity to class-IIa HDACs^32,34^. Due to the short half-life of the [18F]fluoride PET radiotracers, it is highly desirable to design a late-stage labeling site that allows for radiolabeling molecules in a short time with high radiochemical purity and sufficient molar activity. Therefore, we designed a radiochemical route (Figs. 2 and 3) to radiolabel the TFMO moiety with [18F]fluoride and we extended this novel radiofluorination method to incorporate the [18F]fluoride into TMP195 in a single radiochemical step as shown in Fig. 4. This approach enabled the very desirable one-step radiolabeling reaction thus facilitates straightforward automated routine production of [18F]TFMO based PET tracers in the future. By design this is also a universal labeling site that allows for radiolabeling many TFMO containing molecules without changing the radiolabeling strategy as evidenced by our current work.

The chemical synthesis and radiolabeling reactions of the TFMO-containing molecules are shown in Figs. 2 and 3. We started with the TFMO-containing amides to mimic the class-IIa HDAC inhibitors (Fig. 1). Simple amides were selected for simplicity and ease of synthesis. The chemical synthesis of the TFMO containing amides started by coupling the commercially available 3- or 4-(5-(trifluoromethyl)-1,2,4-oxadiazol-3-yl)benzoic acid (**1** or **2**) with benzylamine in the presence of equivalent amount of 1-[Bis(dimethylamino)methylene]-1*H*-1,2,3-triazolo[4,5-*b*]pyridinium 3-oxid hexafluorophosphate (HATU) and excess of 4-methylmorpholine (NMM) to afford the reference standard TFMO amides: *N*-benzoyl-3 or 4-(5-(trifluoromethyl)-1,2,4-oxadiazol-3-yl)benzamide **3** (meta-isomer) or **4** (para-isomer), respectively. The preparation of the essential precursors (**12** and **13**) for radiochemistry started by heating 3- or 4-cyanobenzoate (**5** or **6**) under reflux with hydroxylamine to afford (*Z*)-3- or 4-(*N'*-hydroxycarbamimidoyl)benzoate (**7** or **8**) in quantitative yields and was then treated with bromodifluoroacetic anhydride (**9**) in pyridine to afford the 3- or 4-(5-(bromodifluoromethyl)-1,2,4-oxadiazol-3-yl)benzoate (**10** or **11**) in 70–80% yield. **10** or **11** were coupled with the benzyl amine similar to the preparation of **3** and **4** to afford the bromo-precursors: *N*-benzoyl-3 or 4-(5-(bromodifluoromethyl)-1,2,4-oxadiazol-3-yl)benzamide (**12** and **13**) in 60–70% yield which sets the stage for subsequent radiolabeling reactions.

The radiochemistry was performed initially by treating the bromodifluoromethyl-precursors **12** or **13** with potassium [^18^F]fluoride (K[^18^F]) to produce the [^18^F]TFMO-containing tracers: **[**^**18**^**F]3** or **[**^**18**^**F]4** with very high radiochemical purity (> 98%), albeit the radiochemical yield was very low (< 0.5%). Remarkably, the use of cesium [^18^F]fluoride (Cs[^18^F]) led to a significantly improved radiochemical yield of 2–5%. Cs[^18^F] has been used previously in halogen-exchange radiochemical reaction and cesium carbonate has been shown to facilitate certain nucleophilic [18F]fluorination reactions^41–43^.

To extend the utility of our new radiochemical design, we also radiolabeled TFMO-containing esters with [^18^F]fluoride using the same strategy. Moreover, we generated the radioactive acids **[**^**18**^**F]1** and **[**^**18**^**F]2** which are expected to be generated in vivo from cleavage of the amide bond of the class-IIa HDAC inhibitors (i.e. [^18^F]TMP195) by the metabolizing enzymes. As such, the acid tracers could be useful in determining the metabolic profile of the new tracers in vivo.

The chemical synthesis was performed similar to Fig. 2 above. The commercially available ethyl 3- or 4-cyanobenzoate **14** or **15** was heated under reflux with hydroxylamine to afford (*Z*)-ethyl 3 or 4-(*N'*-hydroxycarbamimidoyl)benzoate (**16**–**17**) in quantitative yield and was then treated with trifluoroacetic anhydride (**18**) to afford the ethyl 3 or 4-(5-(trifluoromethyl)-1,2,4-oxadiazol-3-yl)benzoate (**19**–**20**) in 70–80% yield. Similarly, we synthesized the bromo-precursor analogs ethyl 3- or 4-(5-(bromodifluoromethyl)-1,2,4-oxadiazol-3-yl)benzoate (**20**–**21**) by reacting the oximes **16**–**17** with the bromodifluoroacetic anhydride (**9**) as shown in Fig. 3. Then **20** or **21** was reacted with Cs^18^F at 150 °C in DMSO to successfully generate the desired PET tracer ^**18**^**F-19** or ^**18**^**F-20** in 2–5% radiochemical yield (decay corrected). Hydrolysis of **[**^**18**^**F]19** or **[**^**18**^**F]20** using 1.0 N NaOH afforded the acids **[**^**18**^**F]1** or [^**18**^**F]2** in quantitative yield.

Despite our attempts to improve the radiochemical yield by varying the solvents and temperature as summarized in Table 1, dimethyl sulfoxide (DMSO) at 150–160 °C gave the best radiochemical yield. *N,N*-dimethylacetamide (DMA) gave slightly lower radiochemical yield compared to DMSO; however, the reaction seems to be cleaner (less radioactive and non-radioactive impurities). The use of acetonitrile failed to yield any product. As a result, we selected DMSO as the solvent of choice for subsequent radiolabeling reactions of class-IIa HDAC inhibitors. Notably, no reaction occurred below 145 °C and temperatures > 160 °C produced lower radiochemical yields. We are exploring the use of microwave technology to further improve the radiochemical yield.

**Table 1:** Optimization of the conditions for the [^18^F]-incorporation into the TFMO moiety.

Entry	Base	Solvent	Temp. (°C)	Time	%RCY
1	K_2_CO_3_	DMSO	150	25	< 0.5
2	Cs_2_CO_3_	DMSO	150	25	3–5
3	Cs_2_CO_3_	DMF	150	25	0
4	Cs_2_CO_3_	DMA	150	25	3–5
5	Cs_2_CO_3_	Acetonitrile	150	25	0

Next, we examined the utility of our novel radiochemical design to produce the [^18^F]TFMO-containing class-IIa HDAC inhibitors. Our strategy was designed to incorporate [^18^F]fluoride in the exact location as it appears on the TFMO moiety of class-IIa HDAC inhibitors, thus enabling the radiosynthesis of [^18^F]TMP195 and thus maintaining its exact affinity and specificity to class-IIa HDACs. The radiosynthesis of [^18^F]TMP195, is outlined in Fig. 4. We synthesized the 3-(5-(bromodifluoromethyl)-1,2,4-oxadiazol-3-yl)-*N*-(2-methyl-2-(2-phenyloxazol-4-yl)propyl)benzamide (**23**) similar to the prior synthesis of **12**–**13**. Compound **10** was coupled with the 2-methyl-2-(2-phenyloxazol-4-yl)propan-1-amine (**22**) using HATU and NMM to generate the bromo-precursor, (**23**) in 50–60% yield. Compound **23** was reacted with Cs[^18^F] at 150–160 °C in DMSO for 25 min to successfully generate the desired class-IIa HDAC targeting PET tracer [^18^F]TMP195 in 2–5% radiochemical yield (decay corrected). The radiosynthesis, HPLC purification and formulation of the final radioactive dose was accomplished manually in less than 100 min.

It is critical to note that despite the relatively low radiochemical yield, the radioactivity obtained was sufficient to perform our preclinical imaging studies routinely even when starting with as low as 5.5 GBq (150 mCi). In fact, due to the shorter time of radiosynthesis and dose formulation, it is likely that performing the radiosynthesis starting with high [18F]fluoride dose and using a fully-automated module will significantly improve the amount of the final dose and also expected to further improve the molar activity as was reported for other tracers^40^. Notably, the yield was sufficient for performing our preclinical studies and will also be sufficient to produce a clinical dose for human studies—that is, if a TFMO-based tracer from our ongoing studies becomes available for clinical translation. Notably, the radiosynthesis of [^18^F]TMP195, an inhibitor class-IIa HDACs which is an important target for cancer and brain imaging, described in this publication underscores the significance and novellty of the radiochemical labeling method. Addtionally, it is likely that the radiochemical methods reported herein can be extended to other classes of small trifluoromethyl-containing heterocyclic molecules that appear in inhibitors of highly pursued imaging targets, such as cyclooxygenases (Cox-1 and Cox-2) and estrogen receptors^44–46^.

**Figure 5:**
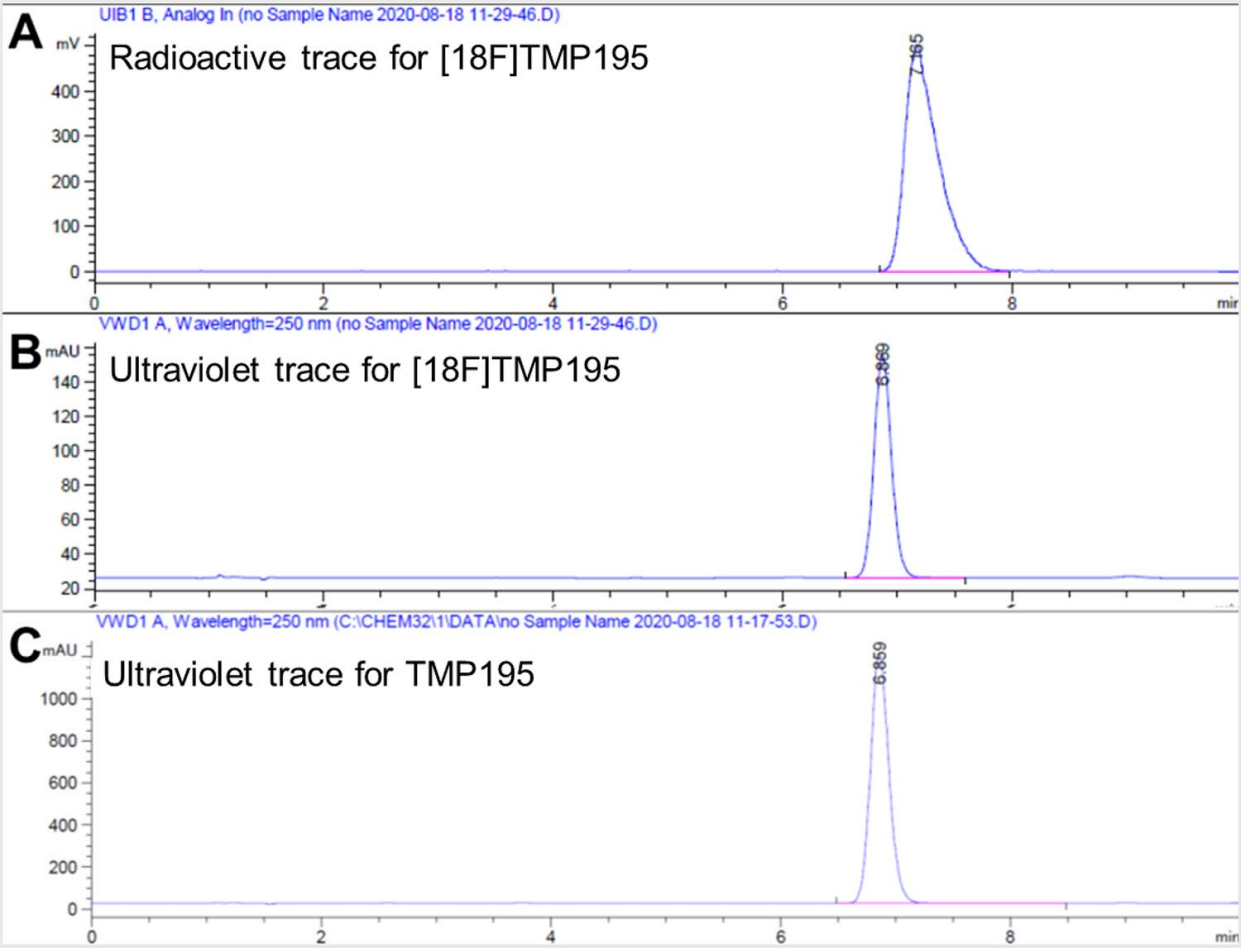
High performance liquid chromatography (HPLC) chromatograms (**a**) radioactive peak of [^18^F]TMP195 detected with radioactivity detector, (**b**) [^18^F]TMP195 associated cold mass was detected with ultraviolet detector and (**c**) TMP195 detected with ultraviolet detector. [^18^F]TMP195 peaks detected by both ultraviolet (250 nm) and radioactive traces were consistent with TMP195 ultraviolet peak, thus confirming the identity of the target tracer.

The new tracer [^18^F]TMP195 was purified using a semi-preparative HPLC system (C18 column with 4.0 ml/min flow rate using 75% acetonitrile/water). The overall radiochemical yield of [^18^F]TMP195 was within 2–5% (end of radiosynthesis, decay-corrected, n = 10) and the radiochemical purity was > 98%. The identity of [^18^F]TMP195 was confirmed by co-injection together with the corresponding cold (unlabeled) molecule using analytical HPLC, as shown in Fig. 5 (chromatograms a and c).

The molar activity of [^18^F]TMP195 was determined from the area under the curve of the tracer that is attributed to the ultraviolet peak in the HPLC chromatogram (Fig. 5, chromatograms B) against a calibration curve pre-prepared with the unlabeled reference standard. The molar activity ranged from 0.33 to 0.49 GBq/umol (8.9–13.4 mCi/umol). Althogh these values are significantly higher than those obtained for [^18^F]‐labeled aryl‐CF_3_ applying [^18^F]CuCF_3_‐based cross‐coupling strategies^36,47^, we expect the molar activity to improve significantly starting with a high dose using a fully automated radiofluorination. Currently, the [^18^F]fluoride is purchased from an outside source and as a result, it undergoes significant decay (> 2 half-lives) before reaching our laboratory which reduces the overall molar activity. Despite the relatively low radiochemical yield, we obtained the tracer in very high purity and the no carrier added radiofluorination led to high molar activity. Furthermore, the labeling bromo-precursor such as compound **23** was easily separable from the reaction mixture using semi-prep HPLC (Fig. 1, Sl). Therefore **23** was not detectable in the final dose and did not confound the molar activity which is a major advantage over the previous reports^39^.

**Figure 6:**
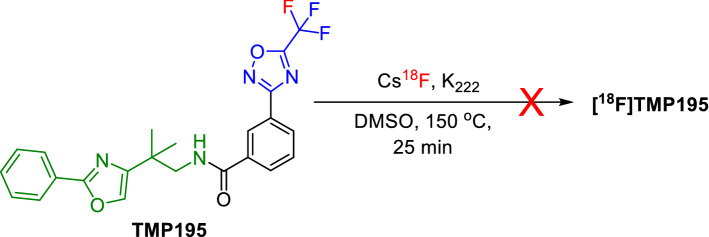
Investigating the ^19^F- to [^18^F] isotopic exchange.

Next, we investigated as to whether the [^18^F]-^19^F isotopic exchange is contributing to the radioactive product, and consequently influencing the molar activity. To rule out this possibility, we performed the radiofluorination under the same conditions above using TMP195 as a starting material as illustrated in Fig. 6. [18F]TMP195 was not obtained and the non-radioactive TMP195 remined unchanged (Figs. 2, Sl) which likely explains the relatively higher molar activity obtained in this work compared to previous reports.

Finally, [18F]TMP195 exhibited poor aqueous solubility which rendered it difficult to formulate for in vivo studies since high concentration of DMSO (~ 50%) was needed to solubilise the tracer. We determined the partition coefficients (LogD) using the octanol/PBS shake flask method^48^. The observed LogD value of [^18^F]TMP195 was > 6.0 which was much higher than the calculated value of cLogD = 5.84 (ChemDraw). In fact, only a background radioactivity was detected in aqueous phase and most of radioactivity retained in the octanol layer. Therefore, other TFMO containing class-IIa HDAC inhibitors are being pursued in our laboratory to identify new PET tracer candidates with improved physiochemical properties and in vitro and in vivo pharmacokinetic profile. The new data will be published in due course.

## Conclusions

In summary, we reported a late-stage radiofluorination of TFMO containing molecules and successful radiosynthesis of class-IIa HDAC targeting PET tracer [^18^F]TMP195. The novel late-stage radiolabeling strategy produced an identical radioactive class-IIa HDAC inhibitor, thus ensuring maintenance of the identical inhibition affinity of TMP195. This strategy is being successfully applied in our laboratory to produce a new generation of [^18^F]TFMO containing class-IIa HDAC inhibitor-based PET tracers. The single radiofluorination step is suitable for straightforward automation and routine production and can produce and formulate the tracer in relatively short period of time. It is also likely that the reported radiochemistry can be extended to other target molecules that contain trifluoromethyl-heterocyclic moiety. Finally. Our novel radiofluorination method reported in this publication will pave the way for the development of TFMO-containing PET tracers for PET imaging of class-IIa HDAC expression in cancer and the disorders of the CNS**.**

## Methods

### General information

Solvents and starting material were obtained from commercial sources and were used as received. High-Performance Liquid Chromatography (HPLC) was performed with a 1260 series pump (Agilent Technologies, Stuttgart, Germany) with a built-in UV detector operated at 250 nm and a radioactivity detector with a single-channel analyzer (labLogic) using a semipreparative C18 reverse-phase column (10 × 250 mm, Phenomenex) and an analytical C18 column (4.6 × 250 mm, ASCENTIS RP-AMIDE, Sigma). An acetonitrile/ammonium acetate buffer (MeCN/NH4OAc: 20 mM) or acetonitrile/water (MeCN/water) solvents with varying composition (solvent systems were developed specific to each compound) was used for quality control analyses at a flow of 1 mL/min. High resolution mass spectroscopy (HRMS) was performed using Agilent 1260HPLC/G6224A-TOF MS. NMR spectroscopy was performed using 400 MHz Bruker instrument.

### Chemical synthesis

TMP195 was prepared similar to the previously described procedures^34^.

#### *General procedure for amine/carboxylic acid coupling reactions (3–4, 12–13 and 23)*

The acid (1.0 eq) and HATU (1.2 eq.) in DMF (1.0–3.0 mL) were stirred for 15 min followed by simultaneous addition of the amine (1.2 eq) and NMM (excess: ~ 1.0 mL). The reaction continued for 3 h. The DMF was removed under vacuum and the residue was purified by column chromatography followed by trituration in cold pentane to afford the final product in 60–80% yield.

#### General procedure for synthesis of (N′-Hydroxycarbamimidoyl)benzoic acid (7–8 and 16–17)

To the nitrile (1.0 g) in ethanol (30 mL) was added first hydroxylamine hydrochloric acid (1.0 g) dissolved in water (8.0 mL) followed by sodium carbonate (1.2 g) dissolved in water (12.0 mL). The mixture was heated under reflux for 4 h. Ethanol was removed under reduced pressure and the residue was diluted with water, acidified with 10% HCl to pH ∼ 3, and filtrated, then washed with water and dried under reduced pressure to afford compound (N′-hydroxycarbamimidoyl)benzoic acid in 50–80% yield.

#### General synthesis of the bromodifluorooxadiazoles (bromo-Precursors: 10–11, 20–21 and 23)

Bromodifluoroacetic anhydride (2.0 mL) neat was added to N′-hydroxycarbamimidoyl benzoic acid, or benzamide. The reaction mixture heated to 50 °C for 3 h. The volatiles were evaporated. Benzoic acids were filtered, washed with water and dried under vacuum. The crude esters and amides were purified by column chromatography using ethyl acetate/hexanes to afford the bromodifluoro-analogs.

#### General synthesis of the trifluorooxadiazoles (19–20)

The synthesis of the TFMO moiety was performed similar to the synthesis described above for bromodifluorooxadiazole except that neat trifluoroacetic anhydride was used.

### Characterization data for the new compounds

#### *N*-Benzyl-3-(5-(trifluoromethyl)-1,2,4-oxadiazol-3-yl)benzamide (3)

^1^H NMR (CDCl_3,_ 400 MHz) δ 8.80 (s, 1H), 8.30 (d, *J* = 8.6 Hz, 1H), 8.08 (d, *J* = 8.4 Hz, 1H), 7.64 (t, *J* = 8.6 Hz, 1 H), 7.34 (m, 5 H), 6.56 (s, 1H), 4.70 (d, *J* = 8.6 Hz, 2H). ^13^C NMR (CDCl_3,_ 100 MHz) δ 170.00 (t, *J* = 23.9), 168.42, 166.10, 137.82, 135.48, 131.16, 130.54, 129.69, 128.89, 128.07, 127.82, 125.78, 125.56, 107.52 (t, *J* = 217.60), 44.36. ^19^F NMR (CDCl_3,_ 376.5 MHz), δ − 64.73. HRMS: Calculated for C_17_H_13_F_3_N_3_O_2_ [M + H] 348.0954, Found: 348.0950.

#### *N*-Benzyl-4-(5-(trifluoromethyl)-1,2,4-oxadiazol-3-yl)benzamide (4)

^1^H NMR (CDCl_3,_ 400 MHz): δ 8.20 (d, 2H), 7.95 (d, 2H), 7.35 (m, 5 H), 6.5 (s, 1H), 4.69 (d, 2H). ^13^C NMR (CDCl_3,_ 400 MHz) δ 168.46, 166.25, 166.0 (q, *J* = 31.8 Hz), 137.78, 137.75, 128.90, 128.03, 127.99, 127.84, 127.83, 127.69, 116.5 (q, *J* = 196.2 Hz), 44.35. ^19^F NMR (CDCl_3,_ 376.5 MHz) δ − 65.30. HRMS: Calculated for C_17_H_13_F_3_N_3_O_2_ [M + H]^+^ 348.0954, Found: 348.0952.

#### 3-(5-(Bromodifluoromethyl)-1,2,4-oxadiazol-3-yl)benzoic acid (10)

^1^H NMR (DMSO-d6, 400 MHz) δ 8.54 (s, 1H), 8.27 (d, *J* = 6, 1H), 8.19 (d, *J* = 6, 1H), 7.75 (s, 1H). ^13^C NMR (DMSO-d6, 100 MHz):δ 170 (t, *J* = 24), 168.28, 166.77, 133.45, 132.43, 131,79, 130.57, 128.40, 125.45, 107.52 (t, *J* = 215.5). ^19^F NMR (DMSO-d6_,_ 376.5 MHz) δ − 54.01. HRMS: Calculated for C_10_H_5_BrF_2_N_2_O_3_ [M + H]^+^ 318.9524, Found: 318.9528.

#### 4-(5-(Bromodifluoromethyl)-1,2,4-oxadiazol-3-yl)benzoic acid (11)

^1^H NMR (DMSO-d6, 400 MHz):): δ 8.14 (m, 5H). ^13^C NMR (DMSO-d6, 100 MHz): δ170 (t, *J* = 23.8), 168.28, 166.94, 134.60, 130.76, 128.71, 128.12, 107.52 (t, *J* = 215.4). ^19^F NMR (DMSO-d6, 376.5 MHz), δ − 54.01. HRMS: Calculated for C_10_H_5_BrF_2_N_2_O_3_ [M + H]^+^ 318.9524, Found: 318.9529.

#### *N*-benzyl-3-(5-(bromodifluoromethyl)-1,2,4-oxadiazol-3-yl)benzamide (12)

^1^H NMR (CDCl_3_, 400 MHz) δ 8.49 (s, 1H), 8.28 (d, *J* = 8.6 Hz, 1H), 8.08 (d, *J* = 8.4 Hz, 1H), 7.64 (t, *J* = 8.6 Hz, 1 H), 7.34 (m, 5 H), 6.56 (s, 1H), 4.70 (d, *J* = 8.6 Hz, 2H). ^13^C NMR (CDCl_3_, 100 MHz) δ 170.00 (t, *J* = 23.9), 168.42, 166.10, 137.82, 135.48, 131.16, 130.54, 129.69, 128.89, 128.07, 127.82, 125.78, 125.56, 107.52 (t, *J* = 217.60), 44.36. ^19^F NMR (CDCl_3,_ 376.5 MHz) δ − 51.98. HRMS: Calculated for C_17_H_13_BrF_2_N_3_O_2_ [M + H]^+^: 408.0154, Found: 408.0152.

#### *N*-Benzyl-4-(5-(bromodifluoromethyl)-1,2,4-oxadiazol-3-yl)benzamide (13)

^1^H NMR (CDCl_3_, 400 MHz)) δ 8.20 (d, *J* = 8.6 Hz, 2H), 7.95 (d, *J* = 8.4 Hz, 2H), 7.35 (m, 5 H), 6.5 (s, 1H), 4.69 (d, *J* = 5.6 Hz, 2H). ^13^C NMR (CDCl_3_, 100 MHz) δ 170.01 (t, *J* = 23.9), 168.33, 166.25, 137.78, 137.65, 128.91, 128.05, 127.99, 127.92, 127.86, 127.77, 107.07 (t, *J* = 217.74), 44.37. ^19^F NMR (CDCl_3,_ 376.5 MHz) δ − 51.97. HRMS: Calculated for C_17_H_13_BrF_2_N_3_O_2_ [M + H]^+^: 408.0154, Found: 408.0153.

#### Ethyl 3-(5-(trifluoromethyl)-1,2,4-oxadiazol-3-yl)benzoate (17)

^1^H NMR (400 MHz, CDCl_3_) δ 8.79 (s, 1H), 8.31 (d, *J* = 5.9 Hz, 1H), 8.28 (d, *J* = 5.9 Hz, 1H), 7.64 (t, *J* = 5.9 Hz, 1H), 4.45 (q, *J* = 5.37, *J* = 10.7 Hz, 2H), 1.45 (t, *J* = 5.34, 3H). ^13^C NMR (CDCl_3_, 100 MHz) δ168.60, 166.98 (q, *J* = 31, *J* = 63 Hz), 165.50, 165.49, 133.18, 131.83, 131.72, 129.33, 128.78, 125.32, 115.51 (q, *J* = 194, *J* = 388 Hz), 61.52, 14.31. ^19^F NMR (CDCl_3,_ 376.5 MHz) δ 65.34. HRMS: Calculated for C_12_H_9_F_3_N_2_O_3_ [M + H]^+^: 287.0644, Found: 287.0638.

#### Ethyl 3-(5-(bromodifluoromethyl)-1,2,4-oxadiazol-3-yl)benzoate (18)

^1^H NMR (CDCl_3_, 400 MHz) δ 8.79 (s, 1H), 8.32 (d, *J* = 5.9 Hz, 1H), 8.30 (d, *J* = 5.9 Hz, 1H), 7.63 (t, *J* = 5.9 Hz, 1H), 4.45 (q, *J* = 5.37, *J* = 10.7 Hz, 2H), 1.45 (t, *J* = 5.34, 3H). ^13^C NMR (CDCl_3_, 100 MHz) δ169.98 (t, *J* = 56.6 Hz), 168.50, 165.56, 133.11, 131.71, 131.68, 129.31, 128.79, 125.53, 107.11 (t, *J* = 215 Hz), 61.52, 14.35. ^19^F NMR (CDCl_3,_ 376.5 MHz) δ 51.95. HRMS: Calculated for C_12_H_10_BrF_2_N_2_O_3_ [M + H]^+^: 346.9843, Found: 346.9837.

#### 3-(5-(Bromodifluoromethyl)-1,2,4-oxadiazol-3-yl)-*N*-(2-methyl-2-(2-phenyloxazol-4-yl)propyl)benzamide (23)

^1^H NMR (CDCl_3_, 400 MHz) δ 8.66 (s, 1H) 8.2 (m, 5 H), 7.65 (t, *J* = 6.6 Hz, 1H), 7.52 (s, 1H), 7.45 (m, 3H), 3.66 (d, *J* = 4.0 Hz, 2H), 1.50 (s, 6H). ^13^C NMR (CDCl_3_, 100 MHz) δ169.92 (t, *J* = 33.4 Hz), 168.62, 166.15, 161.81, 149.14, 136.18, 133.09, 131.16, 131.09, 130.69,130.45, 129.50, 127.09, 126.49, 125.84, 125.57, 107.13 (t, *J* = 300 Hz), 50.62, 34.33, 25.37.^19^F NMR (CDCl_3,_ 376.5 MHz) δ 51.88. HRMS: Calculated for C_23_H_20_BrF_2_N_4_O_3_ [M + H]^+^: 517.0681, Found: 517.0681.

### Radiochemistry

Recipe for the preparation of kryptofix/K_2_CO_3_ solution: K_2_CO_3_ (25 mg) and kryptofix (100 mg) were dissolved in acetonitrile (15.0 mL) and water (5.0 mL).

Recipe for the preparation of kryptofix/Cs_2_CO_3_ solution: Cs_2_CO_3_ (40 mg) and kryptofix (100 mg) were dissolved in acetonitrile (15.0 mL) and water (5.0 mL).

The solution of [18F] was purchased from NCM USA (Bronx, NY). The [18F] is trapped on a QMA cartridge and then eluted with 1.0–1.2 mL of a solution that contains kryptofix/K_2_CO_3_ or kryptofix/Cs_2_CO_3_ to a V-vial (Wheaton) with 92–96% recovery. The eluted [18]Fluoride from the QMA cartridge was used to calculate the %RCY. The solvent was removed under a stream of Argon at 110 °C. Water residue was removed aziotropically with the addition of acetonitrile (3 × 1.0 mL) and repeated drying under a stream of Argon at 110 °C.

A solution of the bromo-precursor (6–8 mg) in the appropriate solvent (i.e. DMSO) (0.4 mL) was added to the dried K[18F]/kryptofix or Cs[18F]/kryptofix and the mixture was heated at 150 °C for 25 min. The reaction mixture was cooled and passed through a silica gel cartridge (waters, 900 mg) and eluted with 30% methanol in dichloromethane (2.5 mL). After evaporating of the solvent under a stream of argon at 60–80 °C, the residue was redissolved in the appropriate HPLC solvent and purified by semipreparative HPLC.

### Authentication of the radioactive tracers

The radioactive peak was detected with a radioactivity detector co-injected with the relevent authentic cold compound which was detected with ultraviolet detector (250 nm) using analytical HPLC. The retention times for the radiotracers are:

- ^**18**^**F-3** was eluted at 5.93 with 60% acetonitrile/water solution.
- ^**18**^**F-4** was eluted at 6.0 with 60% acetonitrile/water solution.
- ^**18**^**F-19** was eluted at 6.8 with 70% acetonitrile/water solution.
- ^**18**^**F-20** was eluted at 6.2 with 70% acetonitrile/water solution.
- ^**18**^**F-1** was eluted at 1.8 with 50% acetonitrile/water solution.
- ^**18**^**F-2** was eluted at 1.8 with 50% acetonitrile/water solution.

*N*-(2-methyl-2-(2-phenyloxazol-4-yl)propyl)-3-(5-([18F]trifluoromethyl)-1,2,4-oxadiazol-3-yl)benzamide ([^18^F]TMP195) was isolated with 75% acetonitrile/water solution in 17–19 min. The solvent was evaporated under reduced pressure and was redissolved in 20%ethanol/saline for animal injection. Each radioactive product was co-injected with an authentic non-radiolabeled compound into an analytical column to confirm its purity and identity. The radiochemical purity was > 95% for all tracers.

### Partition coefficient (logD)

Log D for **[**^**18**^**F]TMP195** was determined using the method similar to our previous work. We determine the log D by partitioning the tracer between octanol and phosphate buffer at pH 7.4 and measuring the concentration of the tracer in each layer. The radioactivity of each layer is counted using gamma-counter. The partition coefficient (P) is calculated as [radioactivity (cpm/mL) in 1-octanol)]/[(radioactivity (cpm/mL) in phosphate buffer pH 7.4].

### Molar activity

The specific activity was determined from the area under the curve of the tracer that is attributed to the ultraviolet peak in the HPLC chromatogram against a calibration curve pre-prepared with the unlabeled reference standard. Molar activity of our tracers ranged from 0.33 to 0.49 GBq/umol (8.9–13.4 mCi/umol) which is remarkably high for [18F]trifluoromethyl moiety. It is important to note that we are currently purchasing F-18 from a commercial source with significant decay prior to the start of our radiochemical experiment (> 2 half-lives). We expect to obtain significantly higher molar activities for our tracers once our cyclotron becomes operational. Furthermore, automated synthesis is expected to further improve the radiochemical yield and molar activity due to more efficient synthesis and shorter overall production time. Moreover, starting with a higher amount of radioactivity may also further improve the yield and molar activities.

## Supplementary Information

Supplementary Information.
