# Supplementary material for: Novel late-stage radiosynthesis of 5-[18F]-trifluoromethyl-1,2,4-oxadiazole (TFMO) containing molecules for PET imaging
Source: Sci Rep. 2021 May 21;11:10668. doi: 10.1038/s41598-021-90069-x (PMC8139947; doi:10.1038/s41598-021-90069-x)
Supplement: Supplementary file 1 — Supplementary Information. [file 41598_2021_90069_MOESM1_ESM.pdf]

Supporting Information

Novel late-stage radiosynthesis of the 5-[18F]-trifluoromethyl-1,2,4-oxadiazole (TFMO) containing molecules for PET imaging

Nashaat Turkman*, Daxing Liu and Isabella Pirola

Table of Contents

Semi-preparative high performance liquid chromatography (HPLC) purifiction of [18F]TMP195 2

Semi-preparative high performance liquid chromatography (HPLC) purifiction for the 19F to [18F] exchange reaction: 3

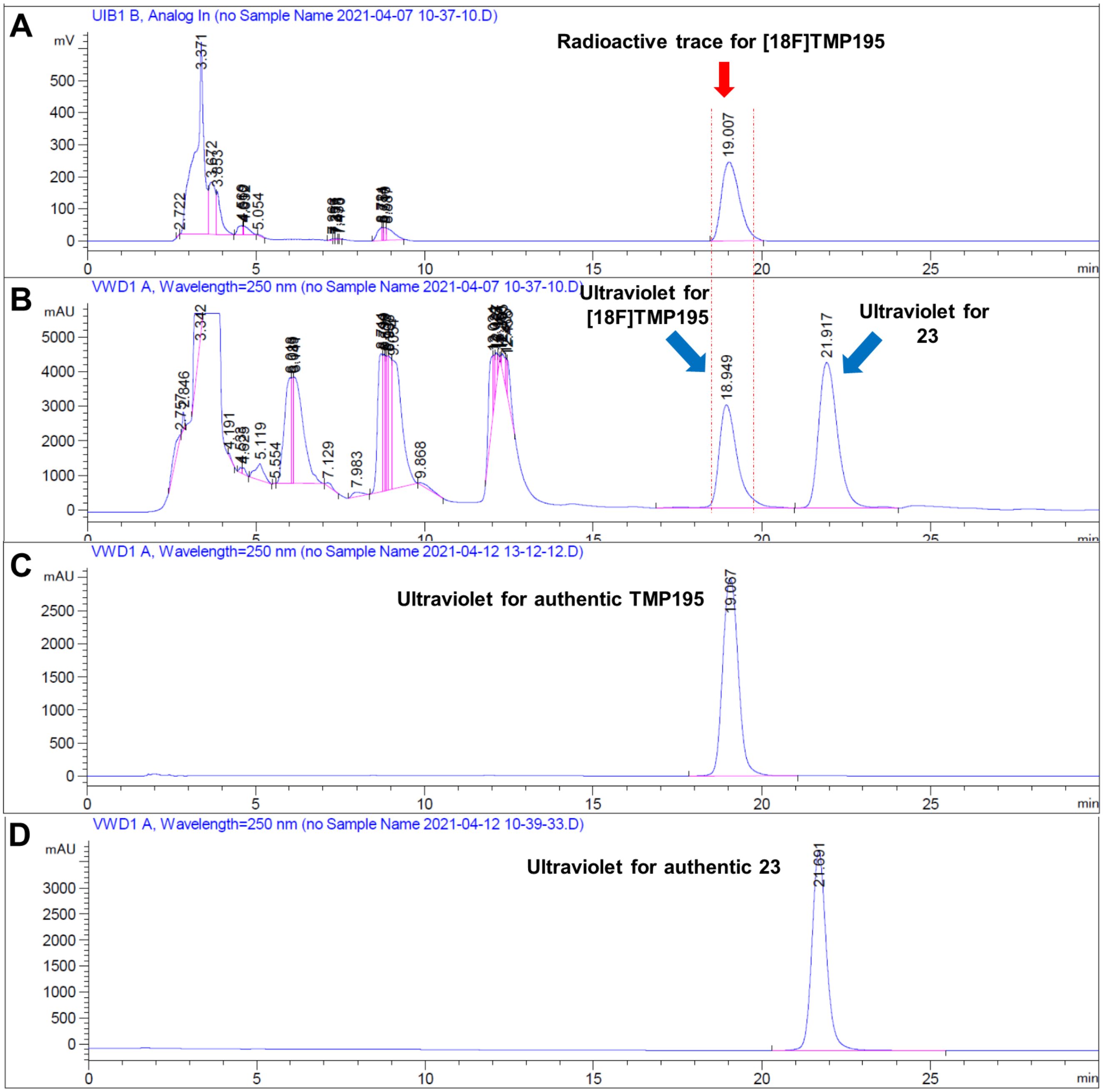

Figure 1. Semi-preparative high performance liquid chromatography (HPLC) purifiction of [18F]TMP195 (a) radioactive peak of [^{18}F]TMP195 in crude mixture detected with radioactivity detector, (b) [^{18}F]TMP195 associated non-radioactive mass and unreacted **23** in the crude reaction mixture were detected with ultraviolet detector, c) authentic TMP195 detected with ultraviolet detector and d) authentic **23** detected with ultraviolet detector. Dashed lines shows start and stop of [^{18}F]TMP195 collection which demeonestrate high radiochemical purity and significant seperation from the labeling precursor **23** and other radioactive peaks.

19F to [18F] exchange reaction: The reaction was condicuted under the same condition used for generating [^{18}F]TMP195 except for starting with the non-radioactive TMP195 (6-8 mg). This expreriment demonestrate that the exchange 19F to [18F] exchange reaction did not occur. TMP195 remained unchanged.

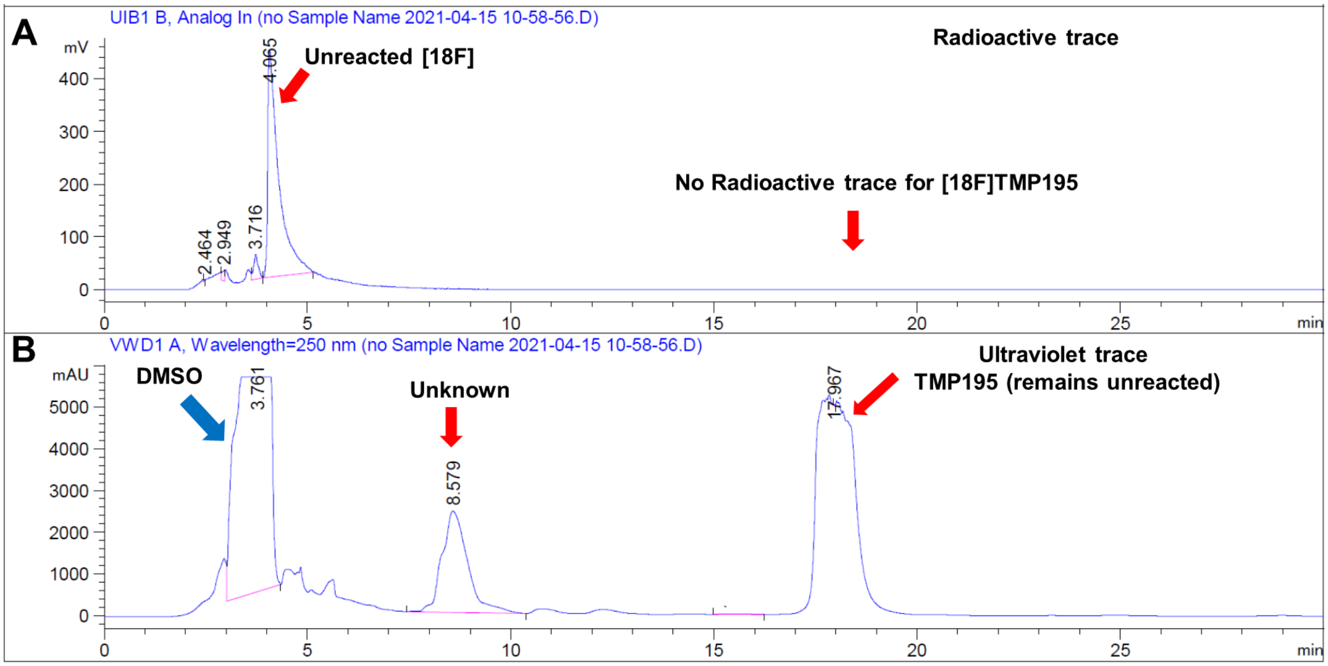

Figure 2. Semi-preparative high performance liquid chromatography (HPLC) purifiction for the 19F to [18F] exchange reaction: (a) radioactive peak of the crude mixture detected with radioactivity detector showed no trace for [^{18}F]TMP195, (b) Ultraviolet associated nonradioactive crude mixture detected with ultraviolet detector demenonstrates that TMP195 remained unreacted in the crude reaction mixture.
